# Treatment of Diabetic Ketoacidosis (DKA)/Hyperglycemic Hyperosmolar State (HHS): Novel Advances in the Management of Hyperglycemic Crises (UK Versus USA)

**DOI:** 10.1007/s11892-017-0857-4

**Published:** 2017-03-31

**Authors:** Ketan K. Dhatariya, Priyathama Vellanki

**Affiliations:** 1 Elsie Bertram Diabetes Centre, Norfolk and Norwich University Hospitals NHS Foundation Trust, Colney Lane, Norwich, Norfolk NR4 7UY UK; 2 Division of Endo, Metabolism & Lipids, Emory University School of Medicine, Atlanta, GA USA; 3 University of East Anglia, Norwich Research Park, Norwich, NR4 7TJ UK

**Keywords:** Diabetic ketoacidosis, Management, Survey, Hyperglycemic hyperosmolar state

## Abstract

**Purpose of Review:**

Diabetic ketoacidosis (DKA) and hyperglycemic hyperosmolar state (HHS) are diabetic emergencies that cause high morbidity and mortality. Their treatment differs in the UK and USA. This review delineates the differences in diagnosis and treatment between the two countries.

**Recent Findings:**

Large-scale studies to determine optimal management of DKA and HHS are lacking. The diagnosis of DKA is based on disease severity in the USA, which differs from the UK. The diagnosis of HHS in the USA is based on total rather than effective osmolality. Unlike the USA, the UK has separate guidelines for DKA and HHS. Treatment of DKA and HHS also differs with respect to timing of fluid and insulin initiation.

**Summary:**

There is considerable overlap but important differences between the UK and USA guidelines for the management of DKA and HHS. Further research needs to be done to delineate a unifying diagnostic and treatment protocol.

## Introduction

Diabetic ketoacidosis (DKA) and hyperglycemic hyperosmolar state (HHS) are hyperglycemic emergencies that continue to account for increased burden of hospitalizations in both the USA [1] and UK [2]. Historically, both DKA and HHS were initially described as one entity but subsequently recognized as separate conditions. Since the advent of insulin, mortality has fallen for DKA and HHS, but the risk remains high. Previous work from the UK and seminal randomized controlled studies performed in the USA by Abbas Kitabchi form the basis of treatment of DKA and HHS. However, only a few of these were randomized studies to guide clinicians on the best way to manage DKA and HHS. Whilst the principles are well known—fluids, insulin, and electrolytes, the questions remain about how much, how fast, etc. This lack of a firm evidence base has led to these small differences in management in both the USA and UK. Additional factors, such as the healthcare environment, also have an impact. In this article, we will discuss the main differences between the USA and UK in the treatment of DKA and HHS.

## Diabetic Ketoacidosis

Prior to the discovery and isolation of insulin in 1922 by Banting and Best, type 1 diabetes was universally fatal within a few months of initial diagnosis. Once mass production was started, the challenge to those early pioneers of insulin treatment was learning how to use this new wonder drug, e.g., how much to give and how often to give it, in order to treat the hyperglycemia without raising the inherent risk of hypoglycemia.

In 1945, Howard Root in Boston described how they had improved the outcomes for people with diabetic ketoacidosis (DKA), reducing mortality to 12% by 1940 and to 1.6% by 1945 using high doses of insulin—giving an average of 83 units within the first 3 h of treatment in 1940 and 216 units by 1945 [3]. They described how in 1945, they used an average of 287 units in the first 24 h, but this ranged from 50 to 1770 units [3]. In Birmingham, UK, high-dose insulin was also being used with similar success—doses varying depending on the degree of consciousness, with those unarousable on admission given doses between 500 and 1400 units per 24 h [4]. DKA remains a medical emergency; over time, mortality has continued to fall but remains a significant risk, especially amongst the young, socially isolated and when care provision is fragmented [5•, 6•]. Overall, the diagnosis and treatment of DKA are very similar in the UK and USA with a few differences. The UK has separate guidelines on the management of DKA [7], while the USA has a position statement on DKA and HHS that was updated in 2009 [8]. The UK guideline differs in several ways from the US position statement.

The concept of low-dose intravenous insulin was established in the late 1960s and early 1970s by teams on both sides of the Atlantic. The UK championed the use of insulin infusions of between 1.2 and 9.6 units per hour at a fixed rate [9–11]. In the USA, Kitabchi et al. used a variety of low-dose insulin regimens, e.g., 0.22 units per kilogram (with a subsequent sliding scale dependent on subsequent glucose concentrations) or 0.33 units per kilogram followed by an infusion of 7 units per hour [12, 13]. These regimens led to a steady reduction in glucose and ketone concentrations at a rate comparable to the higher insulin doses [9–11]. This then led the way to weight-based, fixed-rate intravenous infusion rates [7, 14]. Kitabchi and colleagues went on to do some seminal work looking at the effects of electrolyte disturbances, including the effects of bicarbonate use in DKA. Their significant contributions to the field have been highlighted elsewhere [15].

There are few randomized studies to guide clinicians on the best way to manage DKA. Whilst the principles are well known—fluids, insulin, and electrolytes, the questions remain about how much, how fast, etc. This lack of a firm evidence base has led to these small differences in management. Additional factors, such as the healthcare environment, also have an impact. In the UK, there is the principle of universal health coverage, where payment for healthcare is deducted from income tax and care is provided free at the point of delivery. In the USA, a predominantly insurance-based system exists. In those who have no insurance or minimal health insurance coverage, it would be important to consider ways or providing safe and appropriate treatment that is affordable for the patient and the caregivers. This comparison between the two ways of treating DKA is the focus of this article.

### Differences in Diagnosis

Unlike the USA, the UK has separate guidelines on the management of DKA and HHS [16, 17]. These differ in several ways from the US position statement on hyperglycemic emergencies in adults authored by Kitabchi et al. that was last updated in 2009 [8].

### DKA—Diagnosis

There are differences in the diagnostic criteria for DKA between the UK and the USA (Table 1). There are several potential implications of these differences. The UK criteria suggest that you either have DKA or you do not. But, both documents state that the diagnosis can only be made when all three criteria (the “D,” the “K,” and the “A”) are present. The cornerstone of treatment is administration of fluids and insulin with the endpoint of decreasing ketogenesis.

**Table 1:** UK vs USA diagnostic criteria for DKA

		UK	USA		
			Mild	Moderate	Severe
“D”—a glucose concentration		>11.0 mmol/L (200 mg/dL) or a previous history of diabetes mellitus	>13.9 mmol/L (>250 mg/dL)	>13.9 mmol/L (>250 mg/dL)	>13.9 mmol/L (>250 mg/dL)
“K”—the presence of ketones		>3.0 mmol/L or significant (>2+) on standard urine ketone sticks	Urine or serum ketone positive	Urine or serum ketone positive	Urine or serum ketone positive
“A”—confirmation of an acidosis	pH	<7.3	7.25 to 7.30	7.00 to <7.24	<7.00
	Serum bicarbonate (mmol/L)	<15	15 to 18	10 to <15	<10
	Anion gap	Not applicable	>10	>12	>12

Adapted from [7, 8]

#### The UK Perspective

The UK guideline states that to make a diagnosis of DKA, a prior history of diabetes, regardless of glucose concentrations, although (a glucose >11 mmol/L (200 mg/dL) is specified), is sufficient diagnostic criteria. Due to the availability of testing of 3-beta-hydroxybutyrate testing at the bedside, measurement of serum ketones with a level >3 mmol/L has been suggested as part of the diagnostic criteria for ketoacidosis as opposed to using the urine ketones. Also, the UK guidelines state that using venous blood gas rather than arterial blood gas with a pH <7.3 should be used for diagnosis of acidosis.

There are several advantages to the UK criteria. Approximately 10% of patients with DKA present with euglycemic DKA [18] or with glucose levels below the thresholds set by the US guidelines. This is a condition that is becoming more of an issue with the recognition that it can occur in people taking sodium glucose co-transporter 2 inhibitors [19•] and in pregnancy [20]. Therefore, the emphasis on the history of previous diabetes with a lower glucose threshold than the US criteria allows for detection of euglycemic ketoacidosis. The use of serum rather than urine ketones is advantageous. People with DKA are usually dehydrated, and thus, urine output is low; it may be several hours before urine is produced, further delaying the instigation of appropriate management. Any estimation of urine ketones collected in this way will be an average of the concentration within the urine held in the bladder since the last void. Finally, as the DKA resolves, β-hydroxybutyrate is converted to acetoacetate, which is then excreted into the urine, giving the (false) impression that the condition is taking longer to resolve that it actually is. For these reasons, urine ketone testing is not routinely recommended in the UK guideline. However, because point-of-care, bedside blood ketone meters are not universally available in all hospitals at all times, there is provision made to allow for the occasional use of urine ketones [16, 21••]. The use of venous pH is recommended for the diagnosis of acidosis, because of the data suggesting that the differences between arterial and venous pH are not large enough to change clinical management decisions [22–25]. Furthermore, the anion gap is not used as part of the diagnosis of DKA in the UK. This is in part because a serum chloride is neither routinely reported as part of the blood gas analysis, nor reports of electrolyte concentrations. In addition, the use of 0.9% sodium chloride solution can cause a hyperchloremic metabolic acidosis, and the persistent rise in serum chloride can give the impression to those who are unwary or inexperienced that the resulting high anion gap could be due to the persistent presence of ketones, rather than being due to the fluid resuscitation.

#### The US Perspective

The US guidelines suggest using a glucose threshold of >250 mg/dL (13.9 mmol/L), presence of positive serum and urine ketones with an anion gap, and arterial pH <7.3 to make the diagnosis of DKA. The biggest difference between the UK and the US guideline is the classification of the severity of DKA (Table 1). There are several advantages to categorizing DKA according to severity.

The American Diabetes Association (ADA) consensus guidelines recommend assessment of severity of DKA based on mental status along with the laboratory parameters. While the ADA guidelines acknowledge that approximately 10% of patients with DKA present with lower glucose levels, they emphasize that the key diagnostic feature of DKA is elevated ketonemia. The reasons for dividing DKA presentation into different levels of severity are multifactorial. One of the reasons is due to availability of resources. In the UK, there is the principle of universal health coverage, where payment for healthcare is deducted from income tax and care is provided free at the point of delivery. In the USA, a predominantly insurance-based system exists. In those who have no or minimal health insurance coverage, it would be important to consider ways or providing safe and appropriate treatment that is affordable for the patient and the caregivers. The ADA guidelines also suggest that mental status be used to grade severity. This particular emphasis allows for safer triage of patients presenting to the emergency room to either the intensive care units or step-down units. Further, as per the US guidelines, patients with a bicarbonate level of 18 mmol/L can have mild DKA. This is included to recognize that DKA may be partially treated prior to presentation at the hospital. It should be noted that patients with DKA can have a wide range of acid-base disorders and may have a small anion gap despite increased beta-hydroxybutyrate concentrations [26]. This subset of patients may be erroneously classified as having mild DKA if one was to look for just the anion gap.

For the diagnosis of ketoacidosis, the ADA 2009 guidelines recommend that measurement of ketones by nitroprusside reaction be used because it was more readily available. However, since beta-hydroxybutyrate is the main product of ketogenesis and the nitroprusside reaction does not measure beta-hydroxybutyrate [27], the ADA guidelines suggest measurement of beta-hydroxybutyrate if possible. Further, in the US guidelines, anion gap is used in the diagnostic criteria. Aggressive administration of insulin can cause hyperchloremia and decrease the gap prior to an increase in bicarbonate. Therefore, attention has to be paid to bicarbonate concentrations rather than just the anion gap. The ADA guidelines also recommend the use of arterial pH but state that venous pH can also be used [25, 28, 29].

### Treatment

Both documents agree that the primary treatment should be fluid replacement and that the initial fluid replacement of choice is 0.9% sodium chloride solution. The rates of fluid replacement are similar—the US document advocating 15–20 mL/kg/h (1–1.5 L) in the first hour (regardless of severity) and the UK document 1 L in each of the first 2 h. Both documents agree that phosphate replacement is not needed as the randomized controlled study by Kitabchi et al. did not show differences in outcomes [30]. The rate of insulin infusion is the same in both documents at 0.1 units/kg/h. There are differences in how the insulin infusion rate should be adjusted. The guidelines differ as to the amount and timing of insulin and the use of bicarbonate.

#### UK Perspective

The UK guideline recommends adjustment of insulin infusion depending on the rate of fall of glucose (3.0 mmol/h [54 mg/dL]) and serum ketones (0.5 mmol/h) with a corresponding rise in bicarbonate concentration of 3.0 mmol/L. The UK guideline also incorporates the new evidence to show that the continued use of long-acting basal insulin helps to prevent the rebound hyperglycemia seen when the intravenous insulin is stopped [31].

#### US Perspective

The grading of the severity of DKA directly translates to the relevant treatment regimen. In the USA, Kitabchi et al. performed pioneering studies in the use of low-dose insulin regimens for the treatment of DKA, e.g., 0.22 units per kilogram (with a subsequent sliding scale dependent on subsequent glucose concentrations) or 0.33 units per kilogram followed by an infusion of 7 units per hour [12, 13]. A later study by Umpierrez et al. also showed that frequent subcutaneous insulin injections are just as efficacious as intravenous insulin for the treatment of mild–moderate DKA [32]. Subcutaneous insulin injections can more easily be performed in the general medical units rather than the ICU.

For fluid management, the US guideline suggests the use of 0.45% saline infusion depending on sodium levels. The rate of adjustment of IV insulin differs as well. The US guideline advocates increasing the infusion rate after an hour if the glucose values do not fall by 10%. The UK document does not recommend the use of bicarbonate replacement with the rationale that fluid and insulin replacement alone will be sufficient to raise pH. The US guideline says that bicarbonate should be given when the pH is <6.9 until the pH is >7.0. Even though a prospective randomized study did not show benefit for the use of bicarbonate in severe DKA [33], bicarbonate therapy is recommended when the pH is <6.9 because being acidotic may cause adverse cardiovascular and pulmonary effects [34].

## Hyperglycemic Hyperosmolar State

Unlike DKA, the criteria for diagnosis of hyperglycemic hyperosmolar state (HHS) are not as well defined. It was initially described as a separate entity causing diabetic coma by Dreschfield [35] and Von Frerichs [36]. In the Bradshawe lecture delivered by Dreschfield in 1886, he described three types of diabetic coma. The first one that he described is a gradual coma in older adults (age >40) and in overweight adults without the characteristic acetone breath or acetone in the urine. After this case, several authors described diabetic coma in which polydipsia and polyuria were accompanied by hyperglycemia but without the characteristic Kussmaul breathing seen in DKA [37–39]. Unlike patients with DKA, there was not a presence of ketones or beta-hydroxybutyrate. The full extent of the metabolic derangements seen with HHS was not fully described till the 1950s [40, 41]. In these papers, the authors reported the severe hyperglycemia accompanied by osmotic diuresis but without ketonuria. They also suggested measurement of electrolytes such as sodium and chloride. After this, Gerich et al. [42] and Arieff and Carroll [43] described HHS further and coined the term hyperglycemic hyperosmolar nonketotic coma (HHNK or HONK). A comprehensive history of HHS was described in full detail in a review by Pasquel and Umpierrez [44].

### Diagnosis

HHS occurs mostly in adults and elderly patients and has a higher mortality than DKA with death occurring in 5–16% [45, 46]. The evolution of HHS is over several days to weeks, and the most common presentation is altered mental status [47, 48]. The UK has separate guidelines for the diagnosis of HHS [17]. Due to the lack of randomized controlled trials for the treatment of HHS, the ADA consensus has combined both DKA and HHS [8]. Both statements recommend the assessment of severity at presentation. However, the UK guidelines give specific data cut points to determine the severity of HHS (Table 2). Both recommend evaluation of precipitating causes.

**Table 2:** UK vs USA diagnostic criteria for HHS

		UK	USA
Hyperglycemia		>30 mmol/L (540 mg/dL)	>33.3 mmol/L (600 mg/dL)
Hyperosmolarity		>320 mOsm/kg	>320 mOsm/kg
	Calculation	2 × Na (mmol/L) + glucose (mmol/L) + urea (mmol/L)	2 × Na (meQ/L) + glucose (mg/dL)/18 + blood urea nitrogen (mg/dL)]/2.8
Lack of acidosis	Ketones	Low	Low
	pH	>7.3	>7.3
	Bicarbonate	>15 mmol/L	>20 mmol/L
Mental status changes		Present	Present

Adapted from [7, 8]

The unique distinguishing factor in HHS is the absence of ketones or a low ketone production despite an insulinopenic state. In general, glucose levels in HHS are higher than the ones for DKA. Both the UK guidelines and the ADA suggest similar glucose levels to diagnose HHS. The UK guidelines suggest a cutoff value of glucose >30 mmol/L (540 mg/dL), and the ADA consensus statement suggests a cutoff >33.3 mmol/L (600 mg/dL). In addition to hyperglycemia, patients with HHS present with severe dehydration due to the chronic nature of hyperglycemia. Although there are a subset of patients that present with both DKA and HHS, both groups suggest making a diagnosis of HHS when the pH is greater than 7.3 and the bicarbonate level is greater than 15 mmol/L (UK guidelines) and >20 mmol/L for ADA consensus statement along with minimal ketonemia. The differences in the diagnosis, although minimal, lie in the calculation of osmolality and assessment of severity. The differences are outlined in Table 2.

#### UK Perspective

Both the US and UK guidelines use the cutoff of 320 mOsm/kg for the diagnosis of serum osmolality. This value is based on studies that show that mental status changes occur with serum osmolality >320 mOsm/kg. Osmolality is calculated by the formula [2 × measured Na (meQ/L) + glucose (mg/dL)/18 + blood urea nitrogen (mg/dL)]/2.8] or [2 × measured Na (mmol/L) + glucose (mmol/L) + [urea (mmol/L)]. Blood urea nitrogen is not an effective osmolyte as it can cross membranes without an osmotic effect. The UK guidelines suggest several ways of calculating serum osmolality, which do not include blood urea nitrogen levels. However, due to provider attitudes, it is suggested that it is included in the calculation.

#### US Perspective

Because of its ability to pass freely across plasma membranes, the ADA guidelines recommend calculation of serum osmolality without the inclusion of blood urea nitrogen. Therefore, the patients diagnosed with HHS as per ADA consensus are likely to be more dehydrated prior to receiving intervention for HHS. Again, whether this results in different outcomes compared to the UK guidelines is unclear.

### Treatment

The treatment of HHS, as with DKA, involves correction of dehydration and lowering glucose. Both guidelines recommend careful monitoring of serum osmolality in order to avoid complications of rapid overcorrection. Both guidelines recommend initiation of IV fluids with approximately 1 l of 0.9% saline and aggressive correction of dehydration till osmolality stops declining. Both guidelines suggest maintaining glucose concentrations of 13.9–16.7 mmol/L (250–300 mg/dL in the USA) and 10–15 mmol/L (180–270 mg/dL in the UK). Patients with HHS have depleted potassium stores although less than that in DKA. Both the UK and the ADA consensus statements suggest giving potassium if the concentration is less than 3.3 meQ/L (3.3 mmol/L) and to not replace potassium if the concentration is greater than 5.5 meQ/L (5.5 mmol/L) [8, 17]. The differences in the guidelines are with choice of fluid with respect to sodium concentrations and timing of insulin initiation.

#### UK Perspective

The UK guidelines recommend initial treatment with 1 l of 0.9% saline for the first hour with subsequent adjustment of rate and fluids depending on changes in osmolality (3–8 mOsmol/kg/h) after the first hour for 6 h after presentation with a decrease in glucose of 5 mmol/L/h (90 mg/dL/h). The use of 0.45% saline is not routinely recommended. However, the guidelines do make an exception for the use of 0.45% saline when osmolality does not decline despite adequate fluid administration. Intravenous insulin should be started at weight-based, fixed-rate intravenous insulin at 0.05 U/kg/h once glucose concentrations stop declining with fluid resuscitation when glucose concentrations stop declining with fluid resuscitation.

#### US Perspective

The ADA guidelines recommend initial treatment with 1–1.5 L of 0.9% saline. Unlike the UK, they recommend that 0.45% saline be administered when sodium levels are elevated. Recommendations are made to change fluids according to osmolality. In general, the recommendation is to start intravenous insulin dose at 0.1 U/kg/h once osmolality stops declining. They also suggest doubling the insulin dose if glucose is not falling by 2.8–3.9 mmol/L/h (50–70 mg/dL/h). Once glucose concentrations of 13.9–16.7 mmol/L (250–300 mg/dL) are achieved, the ADA guidelines recommend decreasing insulin dose to 0.02–0.05 U/kg/h.

## Conclusions

In summary, with respect to the management of DKA and HHS, there are wide areas of overlap between the US and UK guidelines. However, there are also equally wide areas where opinions diverge. It is these areas where there are differences of opinion that illustrate the lack of good research to help guide the best treatments for patients. Ultimately, it is fluid, insulin, and potassium replacements, but the questions remain: how much and how fast? For example, there remain questions on which fluid should be used [49] and whether the use of bicarbonate alters the outcomes in those with low pH. Furthermore, more work needs to be done to assess how DKA is diagnosed. In the UK and elsewhere, chloride measurements are not readily available, and therefore, it is impossible to calculate the anion gap. Therefore, if an anion gap cannot be calculated, how do institutions grade severity? Is severity therefore incorrectly based on the presence of 1 or 2 of the D, the K, and the A? If that is the case, then this puts the classification of DKA at risk, and any research using DKA needs to ensure standardization of diagnosis and assessment of severity. Further areas of research also include prospective trial of whether different severities of DKA require different treatments and whether this affects outcomes. There are also no prospective trials for the treatment of HHS. Data is also lacking as to the physiologic basis and outcomes of patients that present with combined DKA and HHS. Finally, both guidelines need to be updated on the recognition and treatment of euglycemic DKA.
